# Improving care and outcomes of inpatients with syndrome of inappropriate antidiuresis (SIAD): a prospective intervention study of intensive endocrine input vs. routine care

**DOI:** 10.1007/s12020-016-1161-9

**Published:** 2016-11-12

**Authors:** Ploutarchos Tzoulis, Helen Carr, Emmanouil Bagkeris, Pierre Marc Bouloux

**Affiliations:** 1 Centre for Neuroendocrinology, Royal Free Campus, University College London Medical School, London, NW3 2QG UK; 2 Centre of Epidemiology and Biostatistics, Institute of Child Health, University College London, London, UK

**Keywords:** Hyponatraemia, Syndrome of inappropriate antidiuretic hormone secretion, Vasopressin, SIADH, Sodium

## Abstract

**Purpose:**

The syndrome of inappropriate antidiuresis is often undertreated with most patients discharged with persistent hyponatraemia. This study tested the hypothesis that an endocrine input is superior to routine care in correcting hyponatraemia and can improve patient outcomes.

**Methods:**

This single-centre prospective-controlled intervention study included inpatients admitted at a UK teaching hospital, with serum sodium ≤ 127 mmol/l, due to syndrome of inappropriate antidiuresis over a 6-month period. The prospective intervention group (18 subjects with mean serum sodium 120.7 mmol/l) received prompt endocrine input, while the historical control group (23 patients with mean serum sodium 124.1 mmol/l) received routine care. The time needed for serum sodium increase ≥ 5 mmol/l was the primary endpoint.

**Results:**

The intervention group achieved serum sodium rise by ≥5 mmol/l in 3.5 vs. 7.1 days in the control group (*P* = 0.005). In the intervention group, the mean total serum sodium increase was 12 mmol/l with only 5.8 % of patients discharged with serum sodium < 130 vs. 6.3 mmol/l increase (*P* < 0.001) and 42.1 % of the subjects discharged with serum sodium < 130 mmol/l in the control group (*P* = 0.012). The mean length of hospital stay in the intervention group (10.9 days) was significantly shorter than in the control group (14.5 days; *P* = 0.004).The inpatient mortality rate was 5.5 % in intervention arm vs. 17.4 % in control arm, but this difference was not statistically significant.

**Conclusions:**

Since the endocrine input improved time for correction of hyponatraemia and shortened length of hospitalisation, widespread provision of endocrine input should be considered.

## Introduction

Hyponatraemia is associated with considerable morbidity and mortality [1–4], and carries a substantial economic burden related to increased hospital resource utilisation, length of hospital stay and risk of readmission [5, 6]. Syndrome of inappropriate antidiuresis (SIAD), the commonest cause of hyponatraemia [7], is often undertreated with recent large observational studies showing that most patients are discharged with persistent hyponatraemia [8–10].

Despite suboptimal standards of care for SIAD, no prospective studies have examined the effect of widespread provision of endocrine input on its management. Also there is paucity of data about whether correcting hyponatraemia can improve patient-related outcomes, such as mortality, length of hospital stay, and readmission rate. The primary hypothesis of our study was that prompt and intensive endocrine input was superior to non-specialised ‘standard’ clinical care in correcting hyponatraemia with the primary endpoint being time to achieve an increase in serum sodium (sNa) by ≥5 mmol/l. Secondary objectives were:

1. to compare the total rise in sNa and the percentage of patients discharged with sNa < 130 mmol/l between intervention and control group
2. to examine the effect of endocrine input on inpatient mortality and length of hospital stay
3. to study whether correction of hyponatraemia improves cognitive function.

## Materials and methods

### Study design

This single-centre prospective-controlled intervention study was conducted in a UK teaching hospital over a 6-month period. During the first 3-month period (1^st^ October 2014–31^st^ December 2014), all patients underwent ‘routine’ care (control group), while in the following 3-month period (1^st^ January 2015–31^st^ March 2015), patients received intensive endocrine input (intervention group). The rationale behind the use of a historical control was that undertaking a randomised controlled trial in a real-life clinical setting was considered impractical since clinicians might often seek expert help from the investigators about patients allocated to the control arm, resulting in possible ‘dilution’ of the control group with patients receiving the intervention. The time required to achieve sNa increase of ≥5 mmol/l was chosen as the primary endpoint since this magnitude of correction can be sufficient to improve symptoms of hyponatraemia [11]. The study received ethical approval from the London-Camden & Islington Research Ethics Committee, and all subjects provided written informed consent before participation.

### Patient selection

All adults with sNa concentration ≤ 127 mmol/l both on hospital admission and on the following day were identified through an automated laboratory system. This cut-off sNa value was selected because previous data from our cohort showed a significant upward inflection in inpatient mortality below that threshold [4]. Among these patients, only subjects who met all essential diagnostic criteria for SIAD, including euvolaemia, hyponatraemia and low serum osmolality with inappropriately raised urine osmolality and sodium, normal adrenocortical reserve, and exclusion of hypothyroidism [12, 13], participated in the study.

Subjects were excluded if they met any of the following exclusion criteria: (1) aged < 18 years old, (2) presence of hypervolaemic hyponatraemia, (3) hypovolaemic hyponatraemia, (4) decompensated chronic liver disease, (5) decompensated heart failure, (6) renal impairment with serum creatinine >200 umol/l or receiving renal replacement therapy, (7) uncontrolled hyperglycaemia with serum glucose >15 mmol/l, (8) pregnancy/breastfeeding, (9) receiving end-of-life care.

Taking into account preliminary data from our cohort indicating a mean time of 5.5 days to reach the primary endpoint of sNa rise ≥5 mmol/l and if standard deviation (SD) for each arm is 1, power sample size was estimated, as 18 patients in each arm, in order to show 20 % difference in the primary endpoint (1.1 day) with 90 % power and 5 % significance level.

### Control group

In real-life clinical practice, the mainstay of SIAD treatment was fluid restriction in combination with discontinuation of offending drugs and treatment of underlying cause. When hyponatraemia was refractory to fluid restriction, some patients were referred to endocrinologists, usually after considerable delay, for consideration of pharmacological therapy. In addition to ‘standard’ clinical care, all patients had full biochemical work-up automatically performed with the attending physicians being notified of the results.

### Intervention group

The investigators, two senior endocrinologists with special interest in hyponatraemia and a Research Nurse, provided regular input on daily basis throughout hospitalisation to the attending medical and nursing team under whose care the patients remained. The intervention reflected best clinical practice [14–16] without the utilisation of any novel diagnostic tests and therapeutic modalities.

Treatment options for SIAD included hypertonic saline, fluid restriction, demeclocycline and tolvaptan. First-line treatment was fluid restriction at a volume of 750–1000 ml/day, apart from cases of severe hyponatraemic encephalopathy requiring urgent correction with intravenous infusion of 1.8 % sodium chloride under close supervision in a high dependency or intensive care unit. In patients not responding to fluid restriction within 48–72 h,second-line treatment, tolvaptan or demeclocycline was prescribed, while urea was not utilised because of lack of availability and absence of local experience in its use. Tolvaptan at a starting dose of 15 mg once per day [17, 18] was used when there was a clinical need for prompt hyponatraemia correction, for example to render a patient fit for chemotherapy or surgery, and in cases with likely short duration of SIAD, for example pneumonia. Demeclocycline at a starting dose of 900 mg per day in divided doses [19] was prescribed in patients with high probability of requiring treatment for longer than 1–2 weeks, such as malignant SIAD.

### Assessment of cognitive function

Taking into account the lack of validated tools to assess symptoms in association with hyponatraemia, Mini-Mental State Examination (MMSE), used extensively to follow the course of cognitive changes over time [20, 21], was performed in all participants of the intervention group at three different time points; on admission, when sNa increased by ≥5 mmol/l from baseline and when sNa was ≥132 mmol/l.

### Statistical analysis

Data were analysed using SPSS (version 21.0, Chicago, IL). Continuous variables were expressed as mean ± SD or percentages. Analysis of variance and Pearson’s *χ* ^2^ test were used to test differences between the intervention and the control group. *P*-values of <0.05 were considered significant.

## Results

### Demographic characteristics

The control group included 23 patients (11 males, 12 females) with a (mean ± SD) age of 77.6 ± 10.7 years compared to the intervention group of 18 subjects (12 males, 6 females) with a (mean ± SD) age of 72.7 ± 10.2 years. There was no statistically significant difference in age and gender distribution across groups.

### Speciality distribution, duration and aetiology of SIAD

There was a wide distribution of patients within different specialities with most patients in both groups being under the care of medical specialities. Chronic hyponatraemia, defined as most recent sNa value measurement within previous 6 months ≤132 mmol/l, was recorded in 34.8 % of cases in the control arm and 44.5 % in the intervention arm (*P* = 0.529). Different aetiologies of SIAD had similar prevalence across groups, as shown in Table 1.

**Table 1:** Classification of cases according to aetiology of SIAD

Aetiology	Control group	Intervention group	*P* value
	*N* = 23 (%)	*N* = 18 (%)	
Pulmonary illness	8 (34.8 %)	6 (33.3 %)	0.923
Idiopathic	6 (26.1 %)	6 (33.3 %)	0.613
Malignancy	5 (21.8 %)	2 (11.1 %)	0.369
Drug-induced	1 (4.3 %)	2 (11.1 %)	0.573
CNS disorder^a^	2 (8.7 %)	1 (5.6 %)	0.702
Various	1 (4.3 %)	1 (5.6 %)	0.859

^a^ Central nervous system pathology

### Baseline biochemical parameters

Serum Na concentration on admission was significantly lower in the intervention arm (120.7 ± 5.5 mmol/l) in comparison with the control arm (124.1 ± 3.1 mmol/l) with a *P* value of 0.017. All other biochemical parameters, apart from serum osmolality, did not differ between two groups, as shown in Table 2.

**Table 2:** Baseline biochemical parameters in both study arms

Biochemical parameters	Control group *N* = 23	Intervention group *N* = 18	*P* value
Serum	Mean ± SD	Mean ± SD	
Na (mmol/l)	124.1 ± 3.1	120.7 ± 5.5	**0.017**
K (mmol/l)	4.4 ± 0.7	4.5 ± 0.7	0.643
Urea (mmol/l)	4.9 ± 2.1	4.3 ± 2.0	0.365
Creatinine (umol/l)	58.7 ± 18.5	59.4 ± 20.5	0.916
Osmolality (mOsm/kg)	259.1 ± 8.0	252 ± 10.2	**0.017**
Urine			
Na (mmol/l)	88.1 ± 48.8	65.3 ± 29.2	0.088
K (mmol/l)	36.6 ± 20.3	36.3 ± 20.2	0.968
Osmolality (mOsm/kg)	445.1 ± 138.0	401.6 ± 146.0	0.333

### Endocrine input

All patients (100 %) in the intervention group received endocrine input compared with 12/23 patients (52.2 %) in the control group (*P* = 0.001). The mean time interval between admission and expert input in the intensive arm was 1.8 days, much shorter than in the ‘routine’ care arm (5.7 days; *P* = 0.007).

### Treatment of SIAD

In the group receiving ‘routine’ clinical care, 26.1 % of patients had no specific treatment for SIAD vs. no untreated cases in the intervention group (*P* = 0.027). The mean number of therapeutic modalities used in the control arm was 1.2, significantly lower than in the intervention arm (1.9; *P* = 0.041). The frequency of utilisation of different therapeutic modalities is illustrated in Table 3.

**Table 3:** Frequency of utilisation of different therapeutic modalities

Treatment modality	Control group	Intervention group	*P* value
	*N* = 23 (%)	*N* = 18 (%)	
Drug discontinuation	5 (21.7 %)	7 (38.9 %)	0.231
Fluid restriction	16 (69.6 %)	18 (100 %)	**0.010**
Tolvaptan	5 (21.7 %)	3 (16.7 %)	0.684
Demeclocycline	2 (8.7 %)	3 (16.7 %)	0.439
Hypertonic saline	1 (4.3 %)	2 (11.1 %)	0.409

### Achievement of primary endpoint

The percentage of patients reaching the primary endpoint of sNa rise ≥5 mmol/l was similar in the intervention group (88.9 %) and in the control group (73.9 %; *P* = 0.230). However, subgroup analysis of control arm indicated that all patients who did not reach the primary endpoint belonged to the subgroup not receiving endocrine input.

The time interval needed for sNa rise ≥5 mmol/l was 3.5 days in the intervention group, almost half the time (7.1 days) required in the control group (*P* = 0.005).

### Rate of hyponatraemia correction

Three days following admission, a further decrease from the baseline in sNa value was observed in 43.5 % of patients in the control arm vs. 5.6 % of subjects in the intervention arm (*P* = 0.007). The intervention group achieved significantly larger magnitudes of sNa correction than the control group, as illustrated in Table 4.

**Table 4:** Correction of sNa 2, 3 and 5 days following admission

Correction of sNa	Control group	Intervention group	*P* value
	*N* = 23	*N* = 18	
2 days			
Correction (mmol/l)^a^	0.3 ± 4.7	1.9 ± 3.5	0.234
Cases with sNa decrease (%)	12 (52.2 %)	4 (22.2 %)	0.051
3 days			
Correction (mmol/l)^a^	0.5 ± 4.7	4.5 ± 3.3	**0.004**
Cases with sNa decrease (%)	10 (43.5 %)	1 (5.6 %)	**0.007**
5 days			
Correction (mmol/l)^a^	1.9 ± 6.2	8.4 ± 3.3	**<0.001**
Cases with sNa decrease (%)	7 (31.8 %)	0	**0.015**

^a^ Mean ± SD

Overly rapid correction of hyponatraemia, defined as sNa increase >12 mmol/l during any 24 h-period or >18 mmol/l during any 48 h-period, was not recorded in the intervention group, while one patient in the control group exceeded the safe limits with sNa increase of 14 mmol/l in the first 24 h following tolvaptan initiation. No cases of osmotic demyelination syndrome were documented.

### sNa at discharge and patient outcomes

**Fig. 1:**
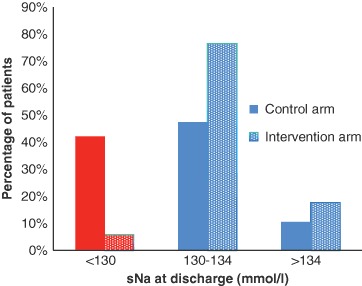
Bar chart illustrating relative frequency distribution of sNa at hospital discharge in both groups. *Solid bars* represent control arm, while *dotted bars* represent intervention arm. *Red bars* show percentage of patients discharged with moderate to severe hyponatraemia (sNa < 130 mmol/l)

As illustrated in Fig. 1, the proportion of patients discharged with moderate to severe hyponatraemia (sNa < 130 mmol/l) in the intervention group (5.8 %) was significantly lower than in the control group (42.1 %; *P* = 0.012). Subgroup analysis of the control arm demonstrated that 30 % of cases receiving endocrine input were discharged with moderate to severe hyponatraemia vs. 55.5 % of cases not receiving endocrine input.

The difference in the mean total sNa increase between intervention arm (12 mmol/l) and control arm (6.3 mmol/l) was statistically highly significant (*P* < 0.001). Further subgroup analysis of the control arm showed higher total sNa rise in patients with endocrine input (9 ± 3.8 mmol/l) compared to patients not receiving endocrine input (4.5 ± 4.8 mmol/l).

As shown in Table 5, the mean length of hospital stay in the intervention group was significantly shorter than in the control group by 3.6 days (*P* = 0.004). The inpatient mortality rate in the intervention group was 5.5 % in comparison to 17.4 % in the control group, but this difference did not reach statistical significance. Among the five fatal cases, two patients, both in the ‘routine’ care group, had persistent severe hyponatraemia at the time of death. The first patient did not receive any specific therapy for SIADH and died with a sNa of 123 mmol/l, while the second patient was not given second-line treatment for SIADH despite failing to respond to fluid restriction and died with a sNa of 122 mmol/l.

**Table 5:** Patient outcomes at discharge

Outcomes	Control arm	Intervention arm	*P* value
	*N* = 23	*N* = 18	
Total sNa rise^a^ (mmol/l)^b^	6.3 ± 0.3	12 ± 6.8	**<0.001**
Inpatient mortality rate	4/23 (17.4 %)	1/18 (5.5 %)	0.250
Length of stay (days)^b^	14.5 ± 7.9	10.9 ± 5.3	**0.004**
Cases discharged on therapy	3/19 (15.8 %)	10/17 (58.8 %)	**0.004**
Readmission rate	5/19 (26.3 %)	4/17 (23.5 %)	0.970

^a^ calculated as [sNa at discharge or death−baseline sNa]

^b^ Mean ± SD

### Effect of hyponatraemia correction on cognitive function

Amongst 18 subjects in the intervention group, 4 patients exhibited severe neurological symptoms, such as acute changes in mental status and confusion, including 2 individuals with Glasgow Coma Score of 12. All these patients showed marked improvement in symptomatology after sNa increase. The remaining patients either had non-specific mild symptoms (9 cases) or seemed asymptomatic (5 cases). Improvement in MMSE score by ≥3 points when sNa reached 132 mmol/l or increased by ≥5 mmol/l was recorded in 38.9 % of subjects in the intervention arm, including 22.2 % of patients with incremental rise of ≥10 points.

## Discussion

This study demonstrated that prompt endocrine input reduced the time required to achieve clinically meaningful sNa increase and led to significantly shorter length of hospital stay.

Several factors may explain the superiority of regular endocrine input to ‘routine’ clinical care in correcting hyponatraemia despite the fact that endocrine input was also provided to almost half of patients in the ‘routine’ care arm. A key factor may be that in the control arm, the average time from presentation to referral for endocrine review was longer than 5 days. Also considerable delay in therapy was frequently observed in the control group, while patients in the intervention group had prompt diagnosis and timely initiation of appropriate treatment for SIAD. In the control arm, around a quarter of patients did not receive any specific treatment for SIAD, whereas there were no untreated cases in the intervention arm. Another main factor differentiating the treatment between groups was the frequency of using fluid restriction, being higher by 30 % in the intervention compared to control arm. Despite the fact that only half of all patients seem to respond to fluid restriction, as shown in Hyponatraemia Registry [10], its implementation in an additional 30 % of cases in the intervention arm might have resulted in significant correction in around 15 % of cases. The increased utilisation of fluid restriction might have made an even more significant contribution to hyponatraemia correction, taking into account that adherence to fluid restriction in the control group was often poor, while in the intervention group, fluid restriction was quite rigorous with a mean oral intake of 800 ml/day and, more importantly, regular patient encouragement in combination with bedside notices, detailed fluid balance charts and removal of excess bedside fluids was used to promote patient compliance. When fluid restriction was ineffective, it was often not followed with an additional therapy in the control arm in contrast to second-line treatment with tolvaptan or demeclocycline being administered in the intervention group, when indicated. Specialist care provision, apart from being effective, was safe without limits for sodium correction being exceeded in any cases and potentially cost-effective with costly pharmacological agents such as tolvaptan being utilised less often than in the control group.

In addition to superiority in correcting hyponatraemia, expert input reduced by 3.6 days of the mean length of hospitalisation. This finding becomes even more noteworthy in light of the unprecedentedly low percentage of patients (5.8 %) discharged with moderate or severe hyponatraemia, much lower than that observed in a recent multicentre UK observational study (23.8 %) [8] and in the Hyponatraemia Registry (43 %) [9]. Moreover, the intervention group had numerically lower inpatient mortality rate (5.5 %) than the control group (17.4 %), but this difference was not statistically significant. Finally, in a considerable proportion of patients, prompt correction of hyponatraemia resulted in rapid improvement of cognitive function.

The main strength of our study was being the first prospective study assessing the effect of expert endocrine input on correction of hyponatraemia and patient-related outcomes. Also this intervention could be readily applicable in everyday clinical practice since the investigators used only tests and therapeutic measures routinely available. Taking into account that ‘routine’ care at our institution included referral to endocrinologists in a large proportion of cases and that the subgroup of the control arm without specialist input achieved much lower sNa increase than the subgroup with endocrine input, our study might underestimate the positive impact of the intervention.

However, this study had a number of limitations. First and foremost, use of historical control instead of randomising subjects to two arms, introduced a potential confounding bias. To minimise the possibility of authors’ bias resulting in shorter length of stay in the intervention group, caring clinical teams responsible for patient care took all clinical decisions such as when a patient should be discharged, with the exception for decisions related to hyponatraemia management. In fact, in a few cases, especially under surgical speciality, the investigators’ input contributed to prolongation rather than shortening of hospitalisation by strongly recommending against hospital discharge, based on high probability of hyponatraemia recurrence and continuing need for close electrolyte monitoring. Also the intervention arm was studied immediately after the control arm in order to ameliorate differences in standards of medical and nursing care, discharge policy, characteristics of hospital population, and clinicians’ prescribing habits. Another possible confounder was the seasonal effect on the distribution of SIAD aetiologies. However, the frequency of pulmonary infection-related SIAD cases was similar across groups since the first arm of the study took place in late autumn and at the beginning of winter, while the second arm in late winter and at the beginning of spring. A further limitation of this study was the small sample size which had adequate power to detect differences in time for correction of hyponatraemia, but it was not powered to identify differences, unless large, in mortality rate and length of stay. The small sample size might also increase the probability of type II statistical error if it introduced confounding through significant differences in the underlying aetiology of SIAD. However the proportion of short duration SIAD cases with a high probability for prompt correction, such as pulmonary infection-related and drug-induced SIAD, was almost identical between intervention and control arm. Additionally, all cases in the intervention arm with drug-induced SIAD, a condition which usually responds very well to drug withdrawal, did not discontinue the, regarded as essential, offending drug, but were treated with fluid restriction. Finally, the generalisability of the positive impact of endocrine input was questionable since it might be highly dependent on the clinical acumen, knowledge and skills of the physicians providing expert input, especially since investigators had extensive experience and expertise in management of SIAD.

This study showed that improving clinical practice led to effective and safe hyponatraemia correction and better patient outcomes. It could be argued that the better standard of care achieved through regular endocrine input might be also met by ‘generalists’. However the consistently suboptimal management of SIAD demonstrated in numerous contemporary studies [8–10] [22] indicated that wider provision of expert input should be considered. To effectively deliver this service, multidisciplinary hyponatraemia teams should be developed. These teams should be led by endocrinologists or other physicians with a special interest in hyponatraemia, such as nephrologists, depending on local expertise. In view of the high prevalence of hyponatraemia, we could consider a 2-tier model of care incorporating electronic alert systems, recently tested with promising results in acute kidney injury [23, 24], with regular involvement of hyponatraemia teams in selected cases.

Our proposed algorithm for optimal SIAD management includes various therapeutic options with individualised treatment decisions being based on several factors, including duration and degree of hyponatraemia, severity of symptoms, the capacity of the nephron to excrete free water, urine osmolality, the safety and efficacy of each treatment modality, cost implications of each therapy and patient compliance [25]. The only treatment modality indicated in patients with severe symptoms related to hyponatraemia, such as seizures, reduced Glasgow Coma Scale and coma, is infusion of hypertonic saline. Treatment with 3 % sodium chloride should commence as bolus of 100 ml over 10–15 min, which should be repeated, if needed, and be followed by intravenous continuous infusion [15]. In the absence of severe hyponatraemic encephalopathy, we aim for an increase in sNa concentration by 4–5 mmol/l/day and not exceeding the limit of 10 mmol/l/day [14] [16] . First-line therapy encompasses treatment of the underlying cause of SIAD and fluid restriction. Restriction of all fluid intake should be titrated according to the Furst^27^ formula using the urine/plasma electrolyte ratio (U/P) = (U_Na_ + U_K_)/(P_Na_ + P_K_), for example 500 ml/day if U/P is 0.5–1.0 and 1000 ml/day if U/P is <0.5 [26]. If urine electrolytes are not readily available, we impose fluid restriction at 750–1000 ml/day. We recommend tolvaptan use as first-line treatment in two groups of SIADH patients; first, if there is a clinical need for prompt correction of hyponatraemia, for example to render a patient fit for chemotherapy or surgery, and second, in cases when fluid restriction is highly unlikely to be effective, evidenced by U/P > 1.0 or urine osmolality > 500 mOsm/kg H_2_O [15]. Tolvaptan should be strongly considered as second-line therapy if a patient has not responded to fluid restriction, defined as sNa increase of ≤3 mmol/l over 48 h. We recommend tolvaptan use only under the supervision of an endocrinologist or nephrologist with patients on tolvaptan maintaining ad libitum fluid intake and not receiving any other concomitant treatment for hyponatraemia. At our institution, we start tolvaptan at a dose of 15 mg for baseline sNa ≥ 125 mmol/l and at 7.5 mg, half the recommended initiating dose, for sNa < 125 mmol/l, since this low starting dose may be associated with lower risk of overly rapid correction, while it retains its efficacy [27–29]. Serum Na concentration should be closely monitored no later than 4–6 h after treatment initiation and at regular 6-h intervals, at least, during the first 24 h of therapy. If serum Na increase exceeds 6 mmol/l at 6 h or 8 mmol/l at any time point between 7 and 18 h following tolvaptan initiation or 10 mmol/l in 24 h, then free water losses are replaced with administration of 5 % dextrose in water at a volume equal to urine output in order to prevent further correction. In cases when reversal of overly rapid correction is warranted, larger volumes of hypotonic fluids are prescribed [16]. Finally and in light of the high cost associated with long-term tolvaptan use, we prescribe demeclocycline at a starting dose of 300 mg three times per day in patients with likely long duration of SIAD, such as malignant SIAD.

Our findings highlight the need for multicentre prospective-controlled studies to examine the impact of specialist input on clinical endpoints such as mortality rate, length of stay, symptoms and readmission rate. Since SIAD represents a potential target for intervention to reduce healthcare expenditures for a large population of inpatients, it is also essential to test the cost-effectiveness of widespread provision of expert input, taking into account on the one hand potential reduction in length of hospitalisation and readmission rate, and on the other hand additional cost related to clinical and nursing time and cost of pharmacological therapies. Finally, studies are warranted to develop tools measuring hyponatraemia-specific symptoms which could be used longitudinally for assessment of symptoms and neurocognitive performance in response to any sodium-correcting therapy.

In conclusion, these preliminary data demonstrated that intensive endocrine input not only was superior to ‘routine’ care in correcting hyponatraemia, but also improved patient-important outcomes such as length of hospital stay and symptoms. If these results could be generalised, provision of systematic endocrine care for patients with SIAD should be widely adopted to improve clinical outcomes and potentially reduce utilisation of hospital resources.
